# The preoperative neutrophil to lymphocyte ratio is a superior indicator of prognosis compared with other inflammatory biomarkers in resectable colorectal cancer

**DOI:** 10.1186/s12885-017-3752-0

**Published:** 2017-11-10

**Authors:** Yongxi Song, Yuchong Yang, Peng Gao, Xiaowan Chen, Dehao Yu, Yingying Xu, Junhua Zhao, Zhenning Wang

**Affiliations:** 1 Department of Surgical Oncology and General Surgery, The First Hospital of China Medical University, 155 North Nanjing Street, Heping District, Shenyang City, 110001 People’s Republic of China; 2 Department of Breast Surgery, The First Hospital of China Medical University, 155 North Nanjing Street, Heping District, Shenyang City, 110001 People’s Republic of China

**Keywords:** Colorectal neoplasms, Lymphocyte to monocyte ratio, Neutrophil to lymphocyte ratio, Platelet to lymphocyte ratio, Prognosis, Prognostic nutritional index, TNM staging

## Abstract

**Background:**

Growing evidence has indicated that some inflammatory markers, including lymphocyte to monocyte ratio (LMR), neutrophil to lymphocyte ratio (NLR), platelet to lymphocyte ratio (PLR), and prognostic nutritional index (PNI), can be used as indicators in the prognosis of colorectal cancer (CRC). However, there is controversy concerning what is the best predictor of prognosis in CRC.

**Methods:**

A cohort of 1744 CRC patients in our institution was analyzed retrospectively. Harrell’s concordance index (c-index) and Bayesian information criterion (BIC) were used to determine the optimal cut-off values of inflammatory markers and compare their predictive capacity. The association of inflammatory markers with overall survival (OS) and cancer-specific survival (CSS) was analyzed using Kaplan-Meier methods with log-rank test, followed by multivariate Cox proportional hazards model.

**Results:**

The multivariate analysis indicated that among these inflammatory markers, NLR (< 2.0 vs. ≥ 2.0) was the only independent prognostic factor for poor OS [hazard ratio (HR) = 0.758, 95% confidence intervals (CI) = 0.598–0.960, *P* = 0.021)] and CSS (HR = 0.738, 95% CI = 0.573–0.950, *P* = 0.018). Among these inflammatory markers, the c-index and BIC value for NLR were maximum and minimum for OS, respectively. In addition, the c-index was higher and the BIC value was smaller in TNM staging combined with NLR compared with the values obtained in TNM staging alone.

**Conclusion:**

NLR is a superior indicator of prognosis compared with LMR, PLR, and PNI in CRC patients, and NLR may serve as an additional indicator based on the current tumor staging system.

**Electronic supplementary material:**

The online version of this article (10.1186/s12885-017-3752-0) contains supplementary material, which is available to authorized users.

## Background

Colorectal cancer (CRC) is the second most commonly diagnosed cancer in women and third in men, with an estimated occurrence of 1.4 million cases and 693,900 deaths in 2012 [1]. At present, TNM staging has been the most commonly used method to predict the prognosis of CRC. However, prognostic heterogeneity still exists in patients with the same TNM stage [2]. Therefore, novel biomarkers are necessary to improve the current tumor staging system and accurately predict the prognosis of CRC.

Recently, growing evidence has indicated that the progression and prognosis of cancer are affected not only by tumor features but also by the inflammatory response of the host [3, 4]. The inflammatory response involves neutrophils, lymphocytes, monocyte, platelets, and acute-phase proteins, including albumin in peripheral blood. The combination of some parameters, including lymphocyte to monocyte ratio (LMR), neutrophil to lymphocyte ratio (NLR), platelet to lymphocyte ratio (PLR), and prognostic nutritional index (PNI), has been used in the prognosis of cancers [5–8], including CRC [9–12]. However, there is controversy about which is the best predictor of prognosis of CRC among these inflammatory biomarkers. On the other hand, to the best of our knowledge, no previous studies have focused on the use of inflammatory biomarkers as a complementary index on the basis of the current TNM staging system. In addition, there are controversies on the optimal cut-off values of these inflammatory biomarkers for predicting prognosis.

In this study, we explored the prognostic value of LMR, NLR, PLR, and PNI in CRC, and compared their ability to predict prognosis. Moreover, we also investigated the optimal cut-off values of these inflammatory biomarkers for predicting prognosis.

## Methods

### Patient cohort

We retrospectively analyzed a cohort of CRC patients who underwent curative resection at the Department of Surgical Oncology at the First Hospital of China Medical University (CMU-SO) between December 2003 and January 2013. Patients without detailed preoperative laboratory data, those who underwent neoadjuvant treatment, and those who used anti-inflammatory medications before surgery were excluded. Finally, 1744 patients were enrolled in the study. Follow-up was completed for all patients until October 2015. The median follow-up was 45.5 months (range of 4–136). Clinical data, including age, sex, clinicopathological features, and preoperative laboratory data, were obtained from the medical records of the patients. The albumin level was obtained using the hepatic function test, and neutrophil, lymphocyte, monocyte, and platelet counts were collected using a routine blood test. PNI was calculated as 10 × albumin level (g/dl) + 0.005 × total lymphocyte count (per mm^3^) [13]. The CRC stage was classified according to the seventh edition of the AJCC/UICC TNM classification system.

### Statistical analysis

Categorical variables were presented as absolute values and percentages and were compared using the chi-square test. Survival rates, including overall survival (OS) and cancer-specific survival (CSS), were analyzed using the Kaplan-Meier method, and differences in variables were compared using log-rank tests. Univariate analysis was used to determine the relationship between the prognostic factors, OS, and CSS. Significant prognostic factors for OS and CSS were included in the multivariate analyses using the Cox proportional hazards model with an enter method.

We assessed the predictive capacity of different categories by measuring discrimination, which is the ability to distinguish between high-risk and low-risk patients. We quantified discrimination and determined the optimal cut-off values for inflammatory biomarkers using Harrell’s concordance index (c-index) [14, 15] and the Bayesian information criterion (BIC) [16]. The maximum c-index value of 1.0 indicates a perfect discrimination. A higher c-index or a smaller BIC value indicated a more desirable model for predicting the outcome.

Statistical analysis was performed using STATA software version 12.0 (Stata Corporation, College Station, TX, USA) and SPSS software version 20.0 (SPSS, Chicago, IL, USA). A *p*-value of less than 0.05 from a two-tailed test was considered statistically significant.

## Results

### Optimal cut-off value of inflammatory biomarkers

The c-index method was used to determine the optimal cut-off values of LMR, NLR, PLR, and PNI for predicting OS. We calculated c-index values for different cut-off values. Our results indicated that the c-index values were maximum for LMR, NLR, PLR, and PNI values of 5.8, 2.0, 134.6, and 46.4, respectively (Table 1). We divided patients into two groups (LMR < 5.8 and ≥ 5.8; NLR < 2.0 and ≥ 2.0; PLR < 134.6 and ≥ 134.6; PNI < 46.4 and ≥ 46.4) for further analysis. There was a minor difference between optimal cut-off values of CSS and those of OS (Table 1) so that the cut-off values of OS were adopted for CSS to maintain consistency and prevent confusion.

**Table 1:** The five greatest c-index values of different cut-off values for LMR, NLR, PLR and PNI

Survival	LMR			NLR			PLR			PNI		
	Cut-off	C-index	N	Cut-off	C-index	N	Cut-off	C-index	N	Cut-off	C-index	N
OS	5.8	0.5461	1192/552	2.0	0.5637	930/814	134.6	0.5540	1015/729	46.4	0.5408	342/1402
	5.3	0.5461	1072/672	2.1	0.5616	1016/728	134.5	0.5536	1014/730	46.3	0.5405	331/1413
	5.2	0.5454	1041/703	2.2	0.5611	1078/666	134.4	0.5533	1012/732	45.5	0.5404	268/1476
	5.4	0.5445	1097/647	1.8	0.5580	770/974	134.3	0.5531	1011/733	45.6	0.5399	283/1461
	5.9	0.5444	1208/536	1.9	0.5578	857/887	130.3	0.5530	954/790	45.9	0.5396	310/1434
CSS	5.2	0.5474	1041/703	2.0	0.5648	930/814	134.6	0.5646	1015/729	46.3	0.5468	331/1413
	5.8	0.5465	1192/552	2.1	0.5613	1016/728	134.5	0.5642	1014/730	45.9	0.5449	310/1434
	5.3	0.5454	1072/672	2.2	0.5611	1078/666	129.4	0.5635	945/799	46.4	0.5447	342/1402
	5.9	0.5453	1208/536	1.9	0.5563	857/887	129.5	0.5635	945/799	45.5	0.5444	268/1476
	5.1	0.5437	998/746	1.8	0.5559	770/974	129.6	0.5635	945/799	45.6	0.5443	283/1461

*Abbreviations*: *CSS* cancer-specific survival, *LMR* lymphocyte to monocyte ratio, *N* number of patients for each group, *NLR* neutrophil to lymphocyte ratio, *OS* overall survival, *PLR* platelet to lymphocyte ratio, *PNI* prognostic nutritional index

### Clinicopathological features and inflammatory biomarkers

Among the 1744 patients evaluated, the median age was 62 (range 13–86); 982 (56.3%) patients were men and 762 (43.7%) were women; 1004 (57.6%) patients were diagnosed with rectal cancer and 740 (42.4%) with colon cancer. The characteristics of the study patients stratified by LMR, NLR, PLR, and PNI are presented in Table 2. The results indicated that LMR was significantly associated with sex, tumor size, tumor location, pT category, and TNM stage (*P* < 0.05); NLR was significantly associated with age, sex, tumor size, tumor differentiation, pT category, and TNM stage (P < 0.05); PLR was significantly associated with sex, tumor size, tumor location, tumor differentiation, pT category, and TNM stage (*P* < 0.05); PNI was significantly associated with age, tumor size, tumor location, tumor differentiation, pT category, and TNM stage (P < 0.05, Table 2).

**Table 2:** Associations of clinicopathological features with LMR, NLR, PLR and PNI in colorectal cancer

Variable	LMR			NLR			PLR			PNI		
	< 5.8	≥ 5.8	*P*	< 2.0	≥ 2.0	*P*	< 134.6	≥ 134.6	*P*	< 46.4	≥ 46.4	*P*
Age(y)			0.086			0.001			0.556			< 0.001
≥ 60	700(58.7)	300(54.3)		499(53.7)	501(61.5)		588(57.9)	421(56.5)		233(68.1)	767(54.7)	
< 60	492(41.3)	252(45.7)		431(46.3)	313(38.5)		427(42.1)	317(43.5)		109(31.9)	635(45.3)	
Gender			< 0.001			< 0.001			< 0.001			0.543
Male	738(61.9)	244(44.2)		481(51.7)	501(61.5)		404(39.8)	371(50.9)		198(57.9)	784(55.9)	
Female	454(38.1)	308(55.8)		449(48.3)	313(38.5)		611(60.2)	358(49.1)		144(42.1)	618(44.1)	
Tumor size (cm)			< 0.001			< 0.001			< 0.001			< 0.001
≥ 4.6	637(53.4)	238(43.1)		395(42.5)	480(59.0)		438(43.2)	437(59.9)		236(69.0)	639(45.6)	
< 4.6	555(46.6)	314(56.9)		535(57.5)	334(41.0)		577(56.8)	292(40.1)		106(31.0)	763(54.4)	
Tumor location			< 0.001			0.073			< 0.001			< 0.001
Colon	544(45.6)	196(35.5)		376(40.4)	364(44.7)		342(33.7)	398(54.6)		193(56.4)	547(39.0)	
Rectum	648(54.4)	356(64.5)		554(59.6)	450(55.3)		673(66.3)	331(45.4)		149(43.6)	855(61.0)	
Differentiation			0.069			0.001			0.001			0.001
Well - moderate	1086(91.1)	517(93.7)		873(93.9)	730(89.7)		952(93.8)	63(6.2)		299(87.4)	1304(93.0)	
Poor - undifferentiated	106(8.9)	35(6.3)		57(6.1)	84(10.3)		651(89.3)	78(10.7)		43(12.6)	98(7.0)	
pT category			0.006			0.019			0.003			< 0.001
T1	30(2.5)	22(4.0)		30(3.2)	22(2.7)		34(3.3)	18(2.5)		3(0.9)	49(3.5)	
T2	188(15.8)	119(21.6)		187(20.1)	120(14.7)		206(20.3)	101(13.9)		39(11.4)	268(19.1)	
T3	470(39.4)	197(35.7)		350(37.6)	317(38.9)		378(37.2)	289(39.6)		157(45.9)	510(36.4)	
T4	504(42.3)	214(38.8)		363(39.0)	355(43.6)		397(39.1)	321(44.0)		143(41.8)	575(41.0)	
pN category			0.497			0.558			0.184			0.412
pN0	689(57.8)	335(60.7)		557(59.9)	467(57.4)		611(60.2)	413(56.7)		194(56.7)	830(59.2)	
pN1	365(30.6)	160(29.0)		273(29.4)	252(31.0)		301(29.7)	224(30.7)		103(30.1)	422(30.1)	
pN2	138(11.6)	57(10.3)		100(10.8)	95(11.7)		103(10.1)	92(12.6)		45(13.2)	150(10.7)	
Distant metastasis			0.130			0.947			0.577			0.337
Negative	1161(97.4)	544(98.6)		909(97.7)	796(97.8)		994(97.9)	711(97.5)		332(97.1)	1373(97.9)	
Positive	31(2.6)	8(1.4)		21(2.3)	18(2.2)		21(2.1)	18(2.5)		10(2.9)	29(2.1)	
TNM stage			0.018			0.026			0.010			0.001
I	176(14.8)	112(20.3)		177(19.0)	111(13.6)		193(19.0)	95(13.0)		32(9.4)	256(18.3)	
II	505(42.4)	221(40.0)		376(40.4)	350(43.0)		415(40.9)	311(42.7)		158(46.2)	568(40.5)	
III	480(40.3)	211(38.2)		356(38.3)	335(41.2)		386(38.0)	305(41.8)		142(41.5)	549(39.2)	
IV	31(2.6)	8(1.4)		21(2.3)	18(2.2)		21(2.1)	18(2.5)		10(2.9)	29(2.1)	

*Abbreviations*: *LMR* lymphocyte to monocyte ratio, *NLR* neutrophil to lymphocyte ratio, *PLR* platelet to lymphocyte ratio, *PNI* prognostic nutritional index

### Prognostic ability of inflammatory biomarkers

**Fig. 1:**
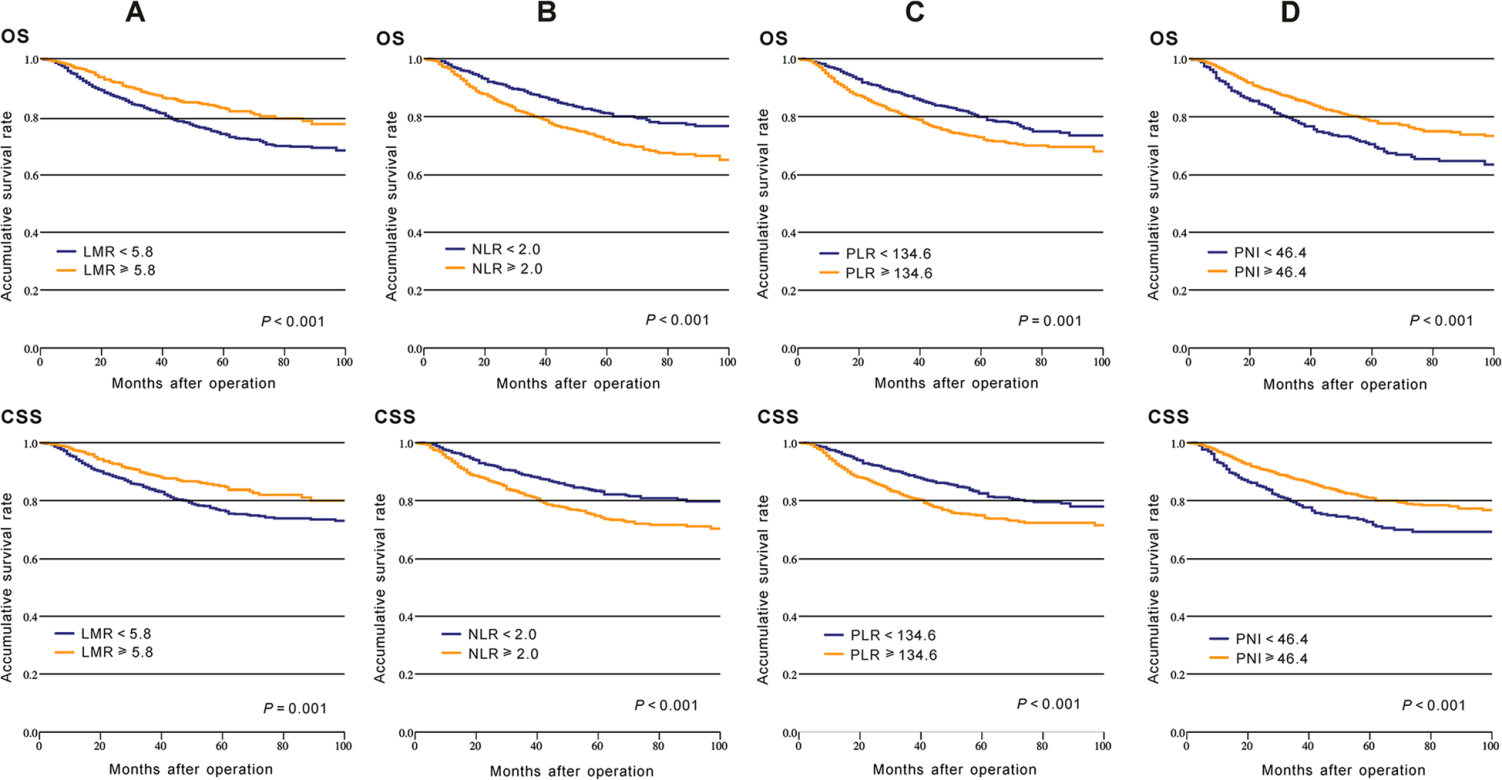
Kaplan-Meier curves of overall survival (OS) and cancer-specific survival (CSS) in CRC patients based on inflammatory biomarkers: **a** LMR; **b** NLR; **c** PLR; **d** PNI

Kaplan-Meier survival analysis with log-rank tests and univariate analysis were performed to evaluate the association between inflammatory biomarkers and prognosis. Our results indicated that LMR, NLR, PLR, and PNI were significantly associated with prognosis of OS and CSS (P < 0.05, Fig. 1, Table 3). However, Cox multivariate analysis indicated that, among the four inflammatory biomarkers, NLR (< 2.0 vs. ≥ 2.0) was the only independent prognostic factor for poor OS (HR = 0.758, 95% CI = 0.598–0.960, *P* = 0.021) and CSS (HR = 0.738, 95% CI = 0.573–0.950, *P* = 0.018, Table 3). Moreover, we regarded LMR, NLR, PLR, and PNI as continuous variables and evaluated the association between these variables and prognosis. The result was similar to that in which inflammatory biomarkers were regarded as dichotomous variables (Additional file 1).

**Table 3:** Univariate and multivariate survival analyses of OS and CSS in patients with colorectal cancer

Variable	Overall survival				Cancer-Specific Survival			
	Univariate		Multivariate		Univariate		Multivariate	
	HR (95% CI)	*P*	HR (95% CI)	*P*	HR (95% CI)	*P*	HR (95% CI)	*P*
Age (y)		0.016		0.005		0.222		
≥60	1		1		1			
<60	0.777 (0.632–0.955)		0.737 (0.597–0.910)		0.873 (0.702–1.086)			
Gender		0.011		0.003		0.084		
Male	1		1		1			
Female	0.766 (0.624–0.940)		0.730 (0.591–0.902)		0.825 (0.663–1.026)			
Tumor Size (cm)		0.397				0.354		
≥4.6	1				1			
<4.6	0.917 (0.751–1.120)				0.904 (0.729–1.120)			
Tumor location		0.732				0.876		
Colon	1				1			
Rectum	1.036 (0.846–1.268)				1.017 (0.819–1.264)			
Differentiation		<0.001		0.001		<0.001		0.001
Well - moderate	1		1		1		1	
Poor - undifferentiated	2.438 (1.836–3.237)		1.647 (1.233–2.198)		2.621 (1.950–3.524)		1.644 (1.216–2.221)	
pT category		<0.001		<0.001		<0.001		<0.001
T1	1		1		1		1	
T2	1.596 (0.486–5.239)		1.390 (0.423–4.573)		0.996 (0.293–3.382)		0.825 (0.243–2.808)	
T3	4.362 (1.393–13.665)		2.132 (0.677–6.715)		3.725 (1.187–11.691)		1.689 (0.534–5.338)	
T4	6.353 (2.029–19.890)		2.910 (0.924–9.165)		5.670 (1.809–17.765)		2.416 (0.766–7.627)	
pN category		<0.001		<0.001		<0.001		<0.001
pN0	1		1		1		1	
pN1	4.779 (3.702–6.170)		4.132 (3.189–5.354)		5.678 (4.249–7.587)		4.856 (3.622–6.512)	
pN2	11.353 (8.583–15.016)		10.215 (7.648–13.643)		14.388 (10.556–19.610)		11.760 (8.565–16.147)	
Distant metastasis		<0.001		0.001		<0.001		0.001
Negative	1		1		1		1	
Positive	3.690 (2.349–5.797)		2.226 (1.407–3.524)		3.974 (2.497–6.324)		2.200 (1.375–3.522)	
TNM stage		<0.001				<0.001		
I	1				1			
II	2.398 (1.327–4.332)				4.854 (1.944–12.118)			
III	12.092 (6.931–21.097)				27.933 (11.524–67.709)			
IV	20.970 (10.408–42.248)				50.019 (18.648–134.163)			
LMR		<0.001		0.698		0.001		0.663
≥5.8	1		1		1		1	
<5.8	1.565 (1.239–1.976)		1.054 (0.809–1.373)		1.541 (1.201–1.977)		1.064 (0.804–1.409)	
NLR		<0.001		0.021		<0.001		0.018
≥2.0	1		1		1		1	
<2.0	0.621 (0.508–0.760)		0.758 (0.598–0.960)		0.620 (0.499–0.770)		0.738 (0.573–0.950)	
PLR		0.001		0.239		<0.001		0.160
≥134.6	1		1		1		1	
<134.6	0.710 (0.582–0.867)		0.873 (0.697–1.094)		0.652 (0.526–0.807)		0.841 (0.661–1.070)	
PNI		<0.001		0.084		<0.001		0.065
≥46.4	1		1		1		1	
<46.4	1.503 (1.199–1.884)		1.238 (0.972–1.576)		1.552 (1.220–1.975)		1.272 (0.985–1.643)	

*Abbreviations*: *CI* confidence interval, *HR* hazard ratio, *LMR* lymphocyte to monocyte ratio, *NLR* neutrophil to lymphocyte ratio, *PLR* platelet to lymphocyte ratio, *PNI* prognostic nutritional index

### Comparison of the prognostic ability of inflammatory biomarkers

**Fig. 2:**
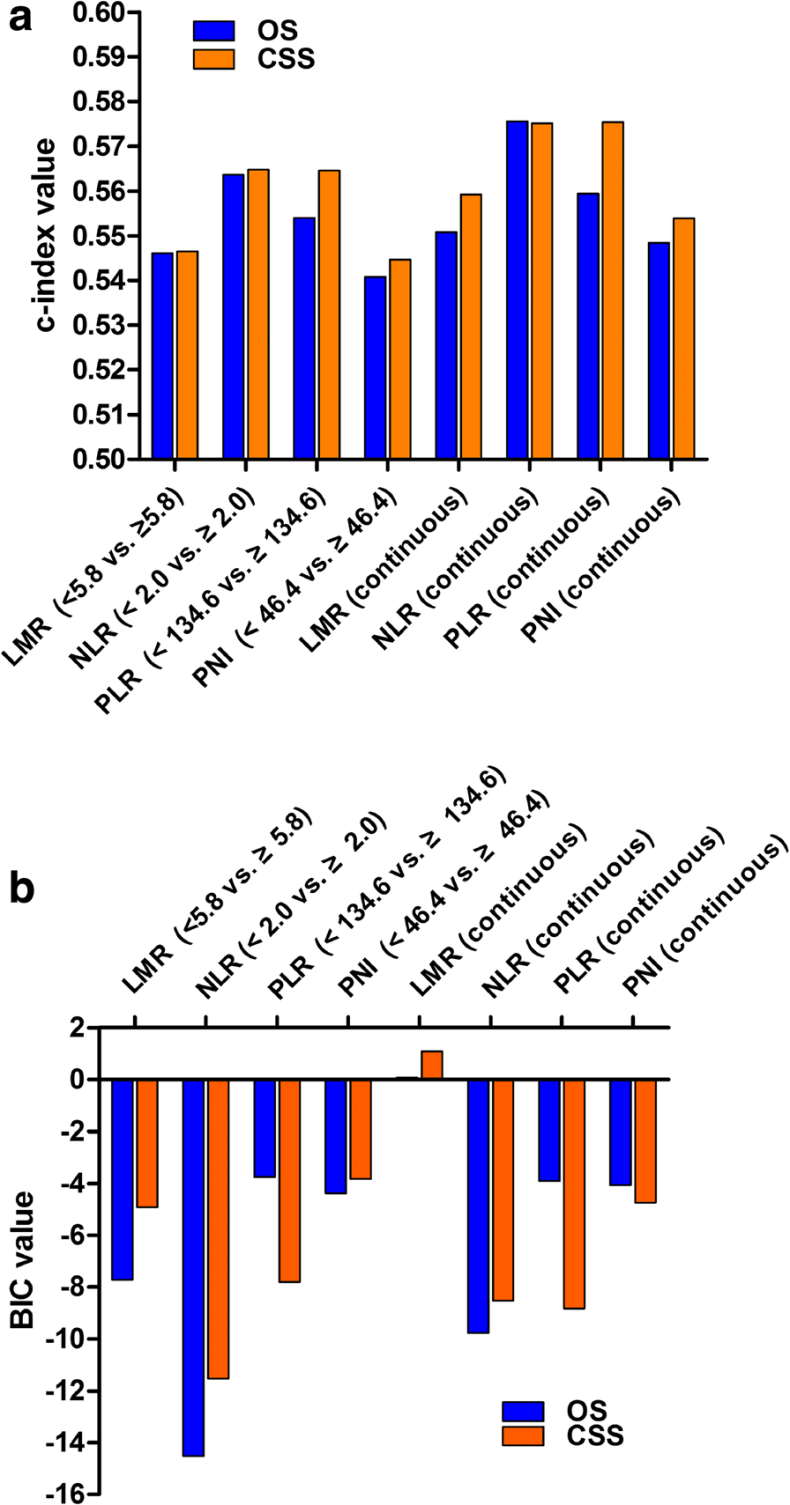
Comparison of the c-index and BIC values for inflammatory biomarkers on overall survival (OS) and cancer-specific survival (CSS): **a** c-index value; **b** BIC values

The c-index and BIC were used to compare the prognostic ability of these four inflammatory biomarkers. When these biomarkers were regarded as binary variables, NLR (< 2.0 vs. ≥ 2.0) had the maximum c-index and minimum BIC for prognosis of both OS and CSS, and when regarded as continuous variables, the NLR presented the maximum c-index and minimum BIC for prognosis of OS (Fig. 2, Additional file 2).

### Evaluation of the prognostic capacity of TNM staging combined with NLR

We calculated the c-index and BIC values of TNM staging combined with NLR (< 2.0 vs. ≥ 2.0) (TNM + NLR) and of TNM staging alone for OS and CSS. The c-index values were greater (OS: 0.7468 vs. 0.7337; CSS: 0.7650 vs. 0.7495) and the BIC values were smaller (OS: −289.723 vs. –271.832; CSS: –299.626 vs. –283.697) in TNM + NLR than those in TNM staging alone.

## Discussion

At present, TNM staging is considered the primary predictor of prognosis. However, this predictor has its limitation because patients at the same TNM stage may have a different prognosis. The introduction of laboratory indexes as additional factors is important for the accurate prediction of prognosis.

Recently, a growing body of evidence suggests that inflammatory biomarkers are associated with clinicopathological features and prognosis in patients with CRC. Accordingly, our results showed that LMR, NLR, PLR, PNI were associated with tumor size, tumor depth, and TNM stage. The results of Kaplan–Meier survival analysis with log-rank tests indicated that these inflammatory biomarkers were significantly associated with prognosis in CRC. However, the actual mechanisms of the association between these inflammatory biomarkers and prognosis in CRC are unclear. There are several potential explanations. First, neutrophils, monocytes, and platelets have been reported to promote tumor development via different mechanisms [17–19], whereas lymphocytes are essential for the elimination of cancer cells [20], and serum albumin is the main plasma protein used to indicate the nutritional status of the host; this may partly explain why elevated NLR, elevated PLR, low LMR, and low PNI were associated with poor prognosis in CRC. Second, elevated NLR and PLR together with low LMR and PNI were significantly associated with advanced tumor features, such as larger tumor size, deeper tumor depth, and advanced TNM stages. Therefore, these variables were associated with the extent of tumor progression, and consequently, affect the survival of CRC patients. Whether they are the causes or consequences of cancer progression remains unknown. Third, the presence of a systemic inflammatory response and/or the poor nutritional status may influence tolerance and compliance with active treatment in cancer patients [21]. However, we did not explore the association between inflammatory biomarkers and active treatment in this study owing to the lack of data; therefore, future studies should evaluate this association.

Controversy still exists concerning the optimal cut-off values of these inflammatory biomarkers for predicting prognosis. In fact, different studies used different cut-off values and different methods to calculate them. Until now, there is no standard method for establishing a universal threshold suitable for every cohort of patients. Some studies used receiver operating characteristic curve analysis (ROC) to dichotomize the inflammatory biomarkers [22–24]. We also used ROC curve analyses to calculate the cut-off values of these four inflammatory biomarkers. Using the 5-year overall survival as an endpoint, the area under the ROC curve for NLR was maximum (See Additional file 3). These results were similar to our results and also indicated NLR had better predictive ability for prognosis compared with other inflammatory biomarkers (See Additional file 3). The results of ROC curve analyses showed that the Youden index was maximum for LMR, NLR, PLR, and PNI values of 5.2, 2.0, 134.6, and 50.8, respectively. We can observe that the cut-off values of LMR and PNI in ROC curve analyses were different from those in c-index analyses. Pencina et al. reported that c-index introduced by Harrell was a natural extension of the ROC curve area to survival analysis, and the method of c-index was calculated based on the survival time and survival state while ROC curve analysis only based on survival state [25]. Therefore, c-index was used to determine cut-off values, in a cohort of 1744 CRC patients in our study. However, the cut-off values identified in this cohort may not apply to other independent cohorts. Therefore, these results need to be confirmed by other studies.

To date, there is no agreement as to which inflammatory biomarkers are the most clinically useful and the best predictors of prognosis in CRC. Some studies showed that NLR was superior to other inflammatory biomarkers as a predictor of prognosis in CRC [23, 26]. Chan et al. [10] reported that LMR was superior to NLR and PLR as a predictor of overall survival; Kwon et al. reported that PLR was a better prognostic serum biomarker than NLR [27], and Park et al. [24] reported that PNI was superior to NLR as a predictor of prognosis in CRC. Our results indicated that either as dichotomous variables or continuous variables, NLR was the only independent prognostic factor for poor OS and CSS among these four inflammatory biomarkers. In addition, we used the c-index and BIC to compare the prognostic capacity of these inflammatory biomarkers, and our results indicated that either as dichotomous variables or continuous variables, NLR had the maximum c-index value and minimum BIC value for OS. These results confirmed that NLR was superior to the other inflammatory biomarkers as a biomarker for predicting prognosis of CRC.

The reason why NLR was superior to other inflammatory biomarkers as a prognostic biomarker in CRC remains unclear. Neutrophils are a major component of leukocyte and can induce several procancer factors, including neutrophil elastase, matrix metalloprotein 9 (MMP9), and vascular endothelial growth factor (VEGF), and therefore are involved in the remodeling of the extracellular matrix and promotion of angiogenesis and tumor development [19, 28, 29]. While lymphocytes are vital components of the host immune system, and lymphocyte infiltration into the tumor is regarded as an anticancer immunologic reaction associated with improved survival [20, 30]. Therefore, NLR may represent a balance between procancer inflammatory reaction and anticancer immune function [9]. We hypothesize that neutrophils and lymphocytes may play more important roles in cancer progression and prognosis than monocytes, platelets, and albumin, which may partly explain our results, although these results need to be confirmed.

Moreover, we first explored the use of NLR as an additional index on the basis of the current TNM staging system. We calculated the c-index and BIC values of TNM + NLR and TNM staging alone. The results showed that TNM + NLR had a greater c-index and a smaller BIC value than TNM staging alone, indicating that TNM + NLR is a better predictive model of prognosis than TNM staging alone. Therefore, NLR may serve as a supplemental index in the current TNM staging system and may increase the prognostic accuracy in CRC.

The present study has several limitations. First, our study was retrospective and uncontrolled. Second, we did not explore the association between prognosis and other inflammatory biomarkers, such as acute-phase proteins, in CRC, owing to the lack of relevant data.

## Conclusion

The preoperative NLR was superior to LMR, PLR, and PNI as a predictor of prognosis, and may serve as an additional index in the current TNM staging system in CRC.

## Additional files

Additional file 1: Univariate and multivariate survival analyses of OS and CSS in patients with colorectal cancer. This table presents the comprehensive results of univariate and multivariate survival analyses of OS and CSS in patients with colorectal cancer. (DOCX 22 kb)

Additional file 2: Comparison of the c-index and BIC values for LMR, NLR, PLR and PNI. This table lists the c-index and BIC values for LMR, NLR, PLR and PNI to make a comparison of these four inflammatory biomarkers. (DOCX 18 kb)

Additional file 3: Receiver operating curve analysis of these four inflammatory biomarkers for 5-year overall survival. This figure shows the ROC curves of LMR, NLR, PLR and PNI along with the area under the ROC curve and *p*-values. (TIFF 953 kb)

## Abbreviations

BIC: Bayesian information criterion
CI: Confidence intervals
c-index: Harrell’s concordance index
CMU-SO: The Department of Surgical Oncology at the First Hospital of China Medical University
CRC: Colorectal cancer
CSS: Cancer-specific survival
HR: Hazard ratio
LMR: Lymphocyte to monocyte ratio
MMP9: Matrix metalloprotein 9
NLR: Neutrophil to lymphocyte ratio
OS: Overall survival
PLR: Platelet to lymphocyte ratio
PNI: Prognostic nutritional index
ROC: Receiver operating characteristic curve analysis
VEGF: Vascular endothelial growth factor
