# Supplementary material for: The preoperative neutrophil to lymphocyte ratio is a superior indicator of prognosis compared with other inflammatory biomarkers in resectable colorectal cancer
Source: BMC Cancer. 2017 Nov 10;17:744. doi: 10.1186/s12885-017-3752-0 (PMC5681757; doi:10.1186/s12885-017-3752-0)
Supplement: Supplementary file 1 — Univariate and multivariate survival analyses of OS and CSS in patients with colorectal cancer. This table presents the comprehensive results of univariate and multivariate survival analyses of OS and CSS in patients with colorectal cancer. (DOCX 22 kb) [file 12885_2017_3752_MOESM1_ESM.docx]

Additional file 1 Univariate and multivariate survival analyses of OS and CSS in patients with colorectal cancer

|  | **Overall survival** | | | |  | **Cancer-Specific Survival** | | | |
| --- | --- | --- | --- | --- | --- | --- | --- | --- | --- |
|  | **Univariate** | | **Multivariate** | |  | **Univariate** | | **Multivariate** | |
| **Variable** | **HR (95% CI)** | ***P*** | **HR (95% CI)** | ***P*** |  | **HR (95% CI)** | **P** | **HR (95% CI)** | ***P*** |
| Age (y) |  | 0.016 |  | 0.005 |  |  | 0.222 |  |  |
| ≥60 | 1 |  | 1 |  |  | 1 |  |  |  |
| <60 | 0.777 (0.632-0.955) |  | 0.739 (0.597-0.915) |  |  | 0.873 (0.702-1.086) |  |  |  |
| Gender |  | 0.011 |  | 0.004 |  |  | 0.084 |  |  |
| Male | 1 |  | 1 |  |  | 1 |  |  |  |
| Female | 0.766 (0.624-0.940) |  | 0.729 (0.590-0.904) |  |  | 0.825 (0.663-1.026) |  |  |  |
| Tumor Size (cm) |  | 0.397 |  |  |  |  | 0.354 |  |  |
| ≥4.6 | 1 |  |  |  |  | 1 |  |  |  |
| <4.6 | 0.917 (0.751-1.120) |  |  |  |  | 0.904 (0.729-1.120) |  |  |  |
| Tumor location |  | 0.732 |  |  |  |  | 0.876 |  |  |
| Colon | 1 |  |  |  |  | 1 |  |  |  |
| Rectum | 1.036 (0.846-1.268) |  |  |  |  | 1.017 (0.819-1.264) |  |  |  |
| Differentiation |  | <0.001 |  | <0.001 |  |  | <0.001 |  | 0.001 |
| Well - moderate | 1 |  | 1 |  |  | 1 |  | 1 |  |
| Poor - undifferentiated | 2.438 (1.836-3.237) |  | 1.684 (1.260-2.249) |  |  | 2.621 (1.950-3.524) |  | 1.677 (1.239-2.269) |  |
| pT category |  | <0.001 |  | <0.001 |  |  | <0.001 |  | <0.001 |
| T1 | 1 |  | 1.000 |  |  | 1 |  | 1 |  |
| T2 | 1.596 (0.486-5.239) |  | 1.327 (0.404-4.360) |  |  | 0.996 (0.293-3.382) |  | 0.785 (0.231-2.668) |  |
| T3 | 4.362 (1.393-13.665) |  | 2.113 (0.671-6.656) |  |  | 3.725 (1.187-11.691) |  | 1.671 (0.529-5.281) |  |
| T4 | 6.353 (2.029-19.890) |  | 2.840 (0.902-8.945) |  |  | 5.670 (1.809-17.765) |  | 2.352 (0.745-7.423) |  |
| pN category |  | <0.001 |  | <0.001 |  |  | <0.001 |  | <0.001 |
| pN0 | 1 |  | 1 |  |  | 1 |  | 1 |  |
| pN1 | 4.779 (3.702-6.170) |  | 4.131 (3.186-5.356) |  |  | 5.678 (4.249-7.587) |  | 4.838 (3.606-6.492) |  |
| pN2 | 11.353 (8.583-15.016) |  | 10.436 (7.809-13.948) |  |  | 14.388 (10.556-19.610) |  | 12.053 (8.769-16.566) |  |
| Distant metastasis |  | <0.001 |  | <0.001 |  |  | <0.001 |  | <0.001 |
| Negative | 1 |  | 1 |  |  | 1 |  | 1 |  |
| Positive | 3.690 (2.349-5.797) |  | 2.355 (1.487-3.730) |  |  | 3.974 (2.497-6.324) |  | 2.327 (1.453-3.726) |  |
| TNM stage |  | <0.001 |  |  |  |  | <0.001 |  |  |
| I | 1 |  |  |  |  | 1 |  |  |  |
| II | 2.398 (1.327-4.332) |  |  |  |  | 4.854 (1.944-12.118) |  |  |  |
| III | 12.092 (6.931-21.097) |  |  |  |  | 27.933 (11.524-67.709) |  |  |  |
| IV | 20.970 (10.408-42.248) |  |  |  |  | 50.019 (18.648-134.163) |  |  |  |
| LMR (continuous) | 0.945 (0.906-0.986) | 0.010 | 1.017 (0.973-1.063) | 0.463 |  | 0.946 (0.903-0.990) | 0.016 | 1.015 (0.968-1.065) | 0.527 |
| NLR (continuous) | 1.154 (1.088-1.225) | <0.001 | 1.154 (1.053-1.264) | 0.002 |  | 1.159 (1.088-1.234) | <0.001 | 1.173 (1.066-1.291) | 0.001 |
| PLR (continuous) | 1.002 (1.001-1.004) | <0.001 | 1.000 (0.998-1.002) | 0.959 |  | 1.003 (1.002-1.005) | <0.001 | 1.000 (0.998-1.002) | 0.905 |
| PNI (continuous) | 0.969 (0.952-0.987) | 0.001 | 0.983 (0.961-1.005) | 0.130 |  | 0.966 (0.948-0.985) | <0.001 | 0.979 (0.957-1.003) | 0.085 |

Abbreviations, CI: confidence interval; HR: hazard ratio; LMR: lymphocyte to monocyte ratio; NLR: neutrophil to lymphocyte ratio; PLR: platelet to lymphocyte ratio;

PNI: prognostic nutritional index.
