# Supplementary material for: The preoperative neutrophil to lymphocyte ratio is a superior indicator of prognosis compared with other inflammatory biomarkers in resectable colorectal cancer
Source: BMC Cancer. 2017 Nov 10;17:744. doi: 10.1186/s12885-017-3752-0 (PMC5681757; doi:10.1186/s12885-017-3752-0)
Supplement: Supplementary file 2 — Comparison of the c-index and BIC values for LMR, NLR, PLR and PNI. This table lists the c-index and BIC values for LMR, NLR, PLR and PNI to make a comparison of these four inflammatory biomarkers. (DOCX 18 kb) [file 12885_2017_3752_MOESM2_ESM.docx]

Additional file 2 Comparison of the c-index and BIC values for LMR, NLR, PLR and PNI

| **Inflammatory biomarkers** |  |  | **OS** | |  | **CSS** | |
| --- | --- | --- | --- | --- | --- | --- | --- |
|  | **Cut-off** | **N** | **C-index** | **BIC value** |  | **C-index** | **BIC value** |
| LMR | 5.8 | 1192/552 | 0.5461 | -7.7209 |  | 0.5465 | -4.9100 |
| NLR | 2.0 | 930/814 | 0.5637 | -14.5229 |  | 0.5648 | -11.5462 |
| PLR | 134.6 | 1105/729 | 0.5540 | -3.7494 |  | 0.5646 | -7.7999 |
| PNI | 46.4 | 342/1402 | 0.5408 | -4.3857 |  | 0.5447 | -3.8275 |
| LMR (continuous) | - | - | 0.5508 | 0.0757 |  | 0.5592 | 1.0900 |
| NLR (continuous) | - | - | 0.5755 | -9.7803 |  | 0.5751 | -8.5268 |
| PLR (continuous) | - | - | 0.5594 | -3.9039 |  | 0.5754 | -8.8336 |
| PNI (continuous) | - | - | 0.5484 | -4.0697 |  | 0.5539 | -4.7439 |

Abbreviations, BIC: Bayesian information criterion; CSS: cancer-specific survival; LMR: lymphocyte to

monocyte ratio; N: number of patients for each group; NLR: neutrophil to lymphocyte ratio; OS:

overall survival; PLR: platelet to lymphocyte ratio; PNI: prognostic nutritional index.
