# Supplementary figures and images for: The preoperative neutrophil to lymphocyte ratio is a superior indicator of prognosis compared with other inflammatory biomarkers in resectable colorectal cancer
Source: BMC Cancer. 2017 Nov 10;17:744. doi: 10.1186/s12885-017-3752-0 (PMC5681757; doi:10.1186/s12885-017-3752-0)

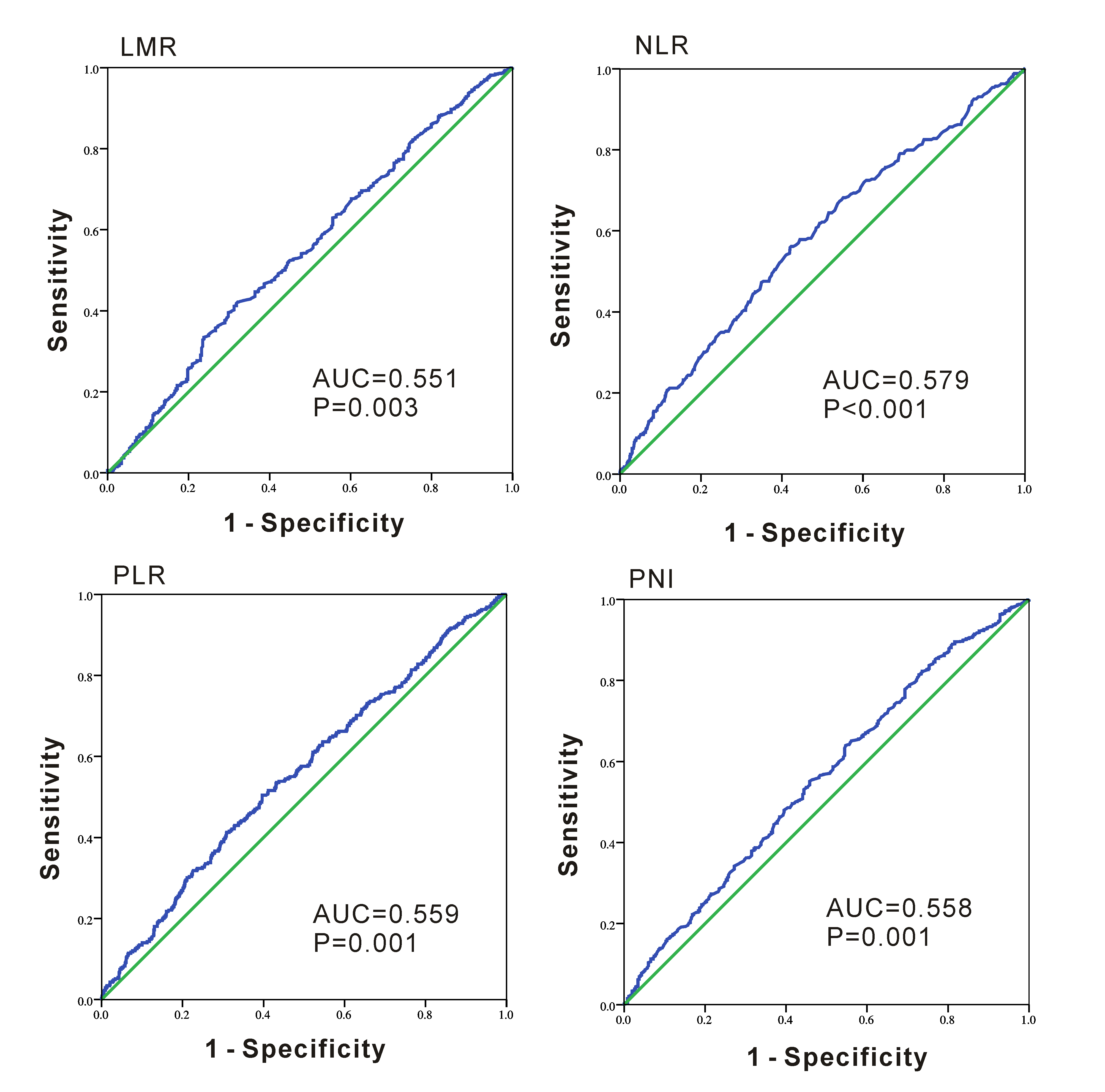

Supplement: Supplementary file 3 — Receiver operating curve analysis of these four inflammatory biomarkers for 5-year overall survival. This figure shows the ROC curves of LMR, NLR, PLR and PNI along with the area under the ROC curve and p-values. (TIFF 953 kb) [file 12885_2017_3752_MOESM3_ESM.tif]
